# Angioarchitectural Factors Associated with Postoperative Cerebral Infarction in Ischemic Moyamoya Disease

**DOI:** 10.3390/brainsci12101270

**Published:** 2022-09-20

**Authors:** Tengfei Yu, Rong Wang, Xun Ye, Chun Zeng, Xiaolin Chen, Yuanli Zhao

**Affiliations:** 1 Department of Neurosurgery, Beijing Tiantan Hospital, Capital Medical University, No. 119 West Nansihuan Road, Fengtai District, Beijing 100070, China; 2 Department of Neurosurgery, Peking University International Hospital, No. 1 Life Park Road, Life Science Park of Zhongguancun, Changping District, Beijing 102206, China

**Keywords:** moyamoya disease, angioarchitectural factor, DSA, direct and indirect revascularization, postoperative cerebral infarction

## Abstract

Objective: To investigate the angioarchitectural factors associated with postoperative cerebral infarction in ischemic moyamoya disease. Methods: Data on patients who underwent surgery for ischemic MMD from 1 October 2015 to 31 October 2020, at Peking University International Hospital were collected and retrospectively analyzed. General conditions such as patient sex, age, site of surgery, preoperative manifestations such as TIA attack and old cerebral infarction, and seven angioarchitectural factors of the MMD based on DSA were selected and measured. Statistical analysis was performed by the Pearson chi-square statistic, analysis of variance (ANOVA), and multifactor logistic regression analysis. Results: Age (OR, 0.969; 95%CI, 0.939–1.000; *p* = 0.049), A1stenosis (OR, 5.843; 95%CI, 1.730–19.732; *p* = 0.004), M1stenosis (OR, 6.206; 95%CI, 2.079–18.526; *p* = 0.001), PCA anomalies (OR, 4.367; 95%CI, 1.452–13.129; *p* = 0.049), Unstable compensation (OR, 5.335; 95%CI, 1.427–19.948; *p* = 0.013), TIA (OR, 4.264; 95%CI, 1.844–9.863; *p* = 0.001), Old cerebral infarction (OR, 2.972; 95%CI, 1.194–7.397; *p* = 0.019). The above seven factors can be used in the regression equation to predict the probability of postoperative cerebral infarction. The prediction accuracy is 90.2%. Conclusions: Age, TIA attack, old cerebral infarction, and five angioarchitectural factors of MMD are strongly associated with postoperative cerebral infarction. Seven factors, including age, TIA attack, old infarction, and four angioarchitectural factors, can be taken to quantify the probability of surgical cerebral infarction in MMD.

## 1. Introduction

Moyamoya disease (MMD), also known as spontaneous occlusion of the circle of Willis, is a rare chronic ischemic cerebrovascular disease that is prevalent in Asian populations [1,2]. It is characterized by the slow progressive narrowing and occlusion of the terminal internal carotid arteries and/or the anterior and middle cerebral arteries and the compensatory formation of a microvascular network at the base of the brain. In recent years, the incidence and prevalence (0.43–2.3/100,000) of MMD in China have gradually increased. There are different stages and subtypes of MMD. It can be divided into ischemic type and effusion type. The classification is based on whether the patient’s first symptom is cerebral hemorrhage or cerebral infarction, and this study is concerned with ischemic type MMD following combined revascularization [3,4,5,6].

Surgical revascularization has been the main treatment for patients with MMD for many years. Overall, the incidence of perioperative stroke during direct, indirect, and combined revascularization procedures ranges from 4.4% to 10%. Among them, postoperative cerebral infarction is our main concern [6,7,8]. Some treatment centers use Suzuki staging for preoperative evaluation, and we found that Suzuki staging can not cover all patients with MMD, especially those with incomplete arterial vascular occlusion [9,10,11,12]. Especially for patients with incomplete vascular occlusion, Suzuki staging does not reflect the degree of their surgical risk. Digital subtraction angiography (DSA), as the most important test for the diagnosis and preoperative evaluation of MMD, provides a comprehensive picture of the vascular architecture in patients with MMD, and it should play a greater role than its purely diagnostic significance [13,14,15,16]. This study will provide a quantifiable and visual reference for the preoperative assessment of surgical risk and thus the selection of the most appropriate treatment modality.

## 2. Materials and Methods

### 2.1. Study Population and Data Collection

Data on patients who underwent surgery for ischemic MMD using CR (combined revascularization) from 1 October 2015 to 31 October 2020, at Peking University International Hospital were collected and retrospectively analyzed. CR group cases refer to the use of a combined surgical approach, which is a combination of direct and indirect revascularization. Direct revascularization refers to superficial temporal artery–middle cerebral artery (STA-MCA) bypass. Indirect revascularization refers to EDAS (encephalo-duro-arteriosynangiosis).

Using the picture archiving and communication system (PACS), basic case information was collected, such as preoperative symptoms, preoperative old infarction, number of TIA episodes, and preoperative imaging, including MRI and DSA. Vascular constructive factors of the surgery side on DSA included local stenosis in the proximal part of the internal carotid artery bifurcation. Localized stenosis of the anterior cerebral artery A1. Localized stenosis of the middle cerebral artery M1. Localized stenosis/occlusion/smoke-like angiogenesis in the posterior cerebral artery. Posterior circulation is compensating for the anterior circulation. Unstable compensation. Compensatory supply of blood from the external carotid artery system to the intracranial. Postoperative complications, such as the presence of cerebral infarction. All variables are based on daily work as well as a literature review [15,16,17,18,19]. Postoperative imaging data, including MRI and CT. All statistical variables were on the same side as the surgical site. If a patient underwent two surgeries, then the two surgeries and their corresponding statistical variables were counted separately.

In the CR group, postoperative cerebral infarction cases were defined as the infarction group, and cases without postoperative cerebral infarction were defined as the no infarction group.

### 2.2. Diagnostic Criteria and Inclusion Criteria

The patients included in the group must have preoperative DSA and MRI and postoperative CT and MRI.

DSA needs to include bilateral P-A and lateral internal carotid or common carotid arteries, P-A and lateral external carotid arteries, and bilateral vertebral or subclavian arteries in the P-A and lateral positions. MRI needs to include at least T1, T2, Flair, and DWI sequences [4,6,8,20].

**Figure 1:**
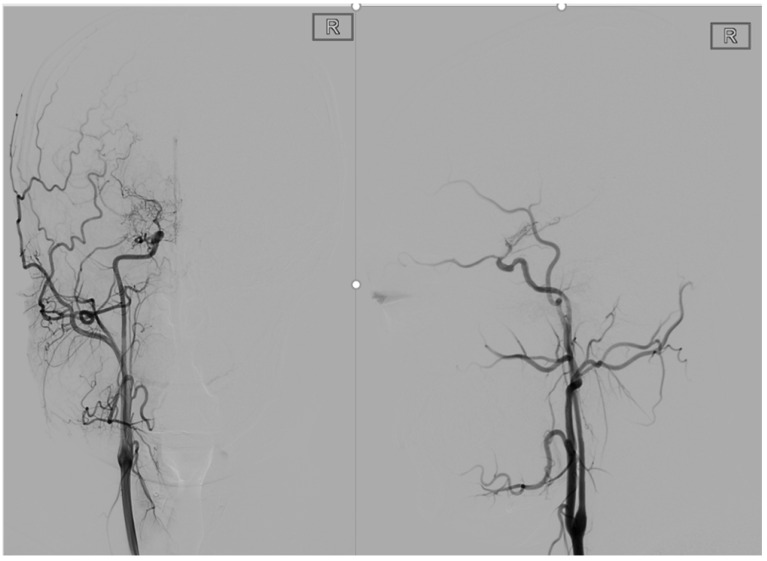
This is a 22-year-old male. These are typical DSA images for MMD. A P-A and lateral view of the internal carotid artery shows occlusion of the right internal carotid artery end with peripheral smoke-like angiogenesis.

Diagnostic criteria for ischemic MMD: 1. DSA demonstrates (1) stenosis or occlusion of the terminal internal carotid artery (ICA) and/or the beginning segment of the anterior cerebral artery (ACA) and/or middle cerebral artery (MCA). (2) An anomalous vascular network at the base of the skull is present in the arterial phase. (3) The above manifestations are bilateral, but the staging of the lesions may be different bilaterally. 3. Combined diseases to be excluded to confirm the diagnosis of MMD (Figure 1).

**Figure 2:**
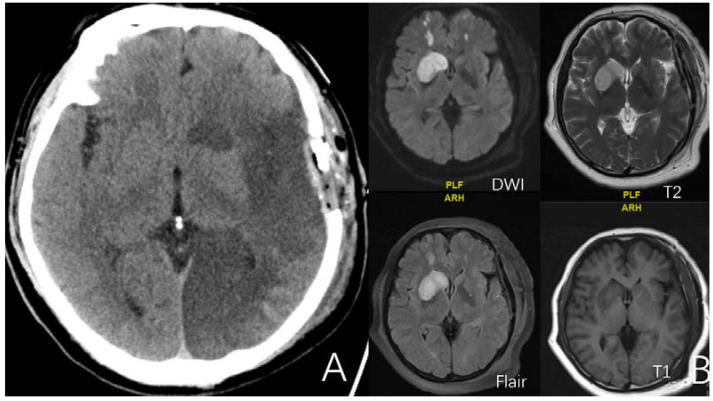
(**A**) is a 52-year-old female. CT showed massive infarct in the left cerebral hemisphere after surgery. (**B**) is a 52-year-old female. Four sequences of MRI exhibit the imaging features of minor cerebral infarction after surgery.

The diagnostic criteria for postoperative cerebral infarction need to meet the following three points at the same time: 1. Symptoms of neurological dysfunction, such as confusion, unfavorable limb movement, language dysfunction, and visual field defects. Head CT performance: one or more round, oval, or rectangular low-density lesions with clear borders and no occupying effect can be seen in the intracranial parenchyma on CT. 3. MRI performance: low signal on T1 sequence, high signal on T2 sequence, high signal on DWI sequence and Flair sequence. In this study, postoperative cerebral infarction was defined as a cerebral infarction within one week after surgery [10,21,22,23,24] (Figure 2).

### 2.3. Data Collection

All data were retrospectively analyzed by two attending physicians using the PACS without any prior information. The final statistics were combined, with a senior physician deciding whether the established criteria were met in case of disagreement.

Twelve influencing factors were included in this study. They are 5 general conditions as well as 7 vascular constructive factors. All factors are assigned numerical values. These values were used in the data statistics and in the calculation of the multifactor regression analysis equation.

#### 2.3.1. Gender 

Male is assigned a value of 1, and female is assigned a value of 2.

#### 2.3.2. Side 

The left side is assigned to 1, and the right side is assigned to 2.

#### 2.3.3. Age 

Taking the integer of the patient’s age at the time of surgery.

#### 2.3.4. TIA

TIA was defined as one or more transient ischemic attacks within 6 weeks prior to surgery. Positive cases are assigned a value of 1, while negative cases are assigned a value of 0.

#### 2.3.5. Old Cerebral Infarction 

**Figure 3:**
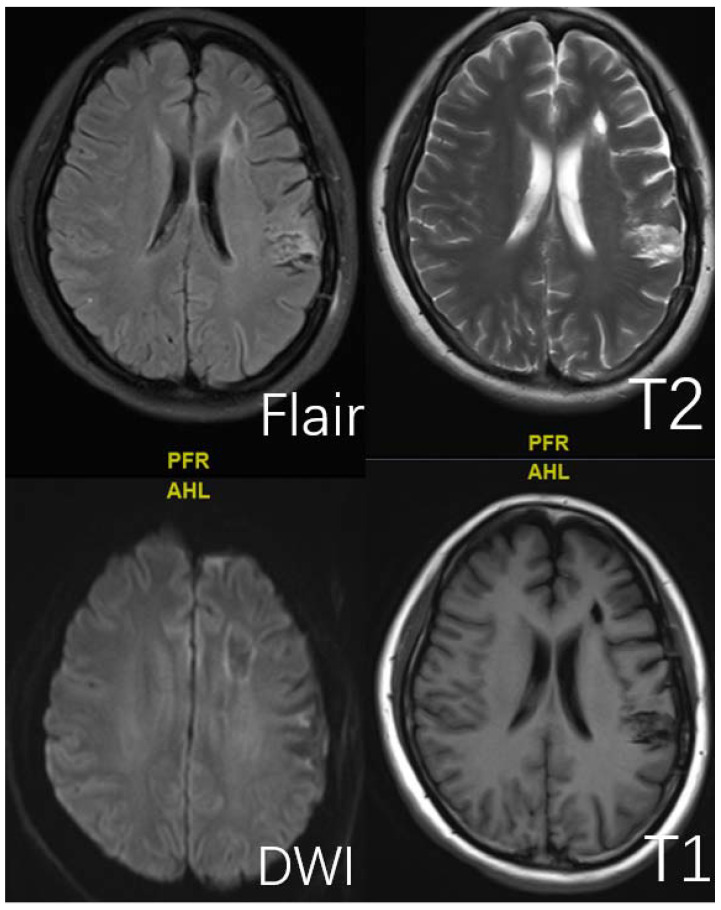
This is a 45-year-old female. Typical presentation of old cerebral infarction on T1, T2, DWI, and Flair sequences.

Old cerebral infarction was defined as a cerebral infarction more than three months prior to surgery. Maximum diameter of infarct lesion ≥10 mm. MRI showed a low signal in the T1 sequence, a high signal in the T2 sequence, a high signal in the Flair sequence, and an isosignal or high signal in the DWI sequence. Positive cases are assigned a value of 1, while negative cases are assigned a value of 0 (Figure 3).

The 7 vascular constructive factors on the side of surgery based on DSA are defined as following sections.

#### 2.3.6. Factor 1 ICA Stenosis

**Figure 4:**
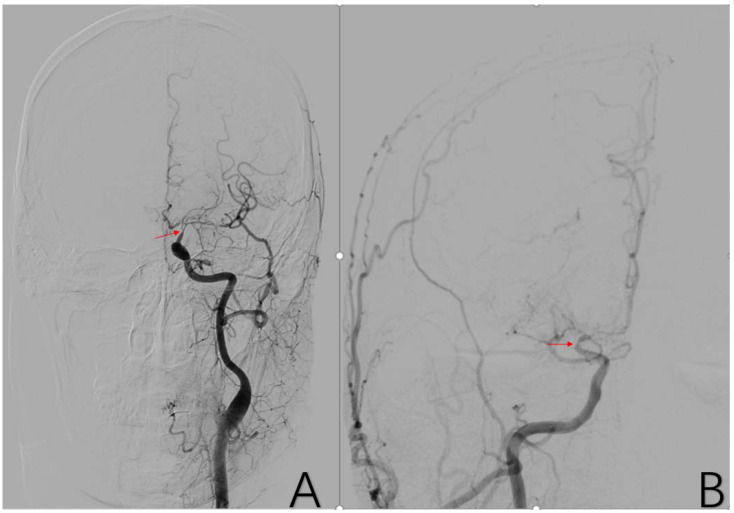
(**A**) is a 48-year-old male. The typical image presentation of internal carotid artery stenosis can be demonstrated on this DSA of the common carotid artery. Additionally, there was a typical presentation of ICA stenosis on the right side. The red arrows in (**A**,**B**) are the tool of the PACS and show the location of ICA stenosis.

The middle cerebral artery and/or anterior cerebral artery distal to the bifurcation of the internal carotid artery remain incompletely occluded, while the proximal part of the bifurcation has segmental stenosis without complete occlusion. Positive cases are assigned a value of 1, while negative cases are assigned a value of 0 (Figure 4).

#### 2.3.7. Factor 2 A1 Stenosis

**Figure 5:**
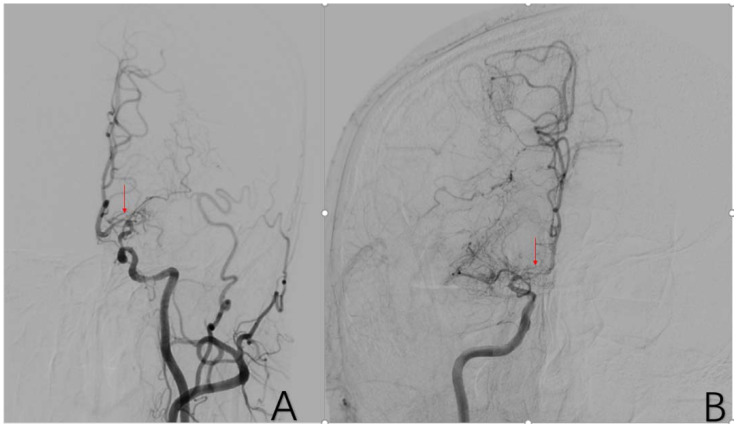
(**A**) is a 48-year-old male. This is a DSA image of his left common carotid artery. (**B**) is a 43-year-old male. This is a DSA image of his right internal carotid artery. The red arrows show the location of A1 stenosis.

Localized stenosis of the beginning segment of the anterior cerebral artery (ACA) with incomplete occlusion of the vessels distal to A1. Positive cases are assigned a value of 1, while negative cases are assigned a value of 0 (Figure 5).

#### 2.3.8. Factor 3 M1 Stenosis

**Figure 6:**
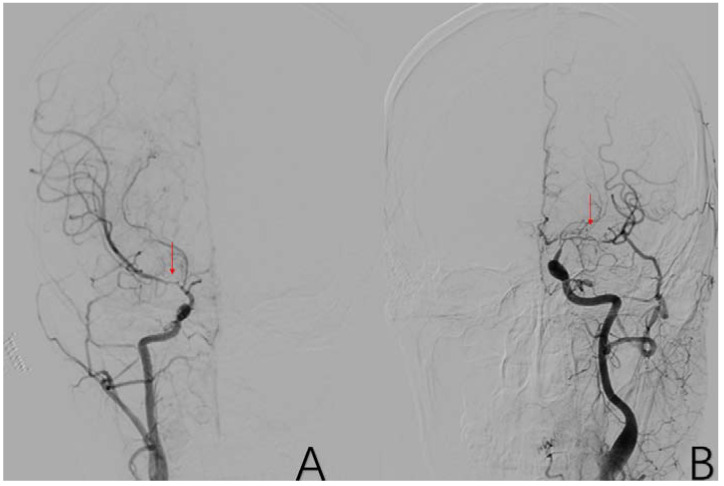
(**A**) is a 45-year-old female. This is a DSA image of his right common carotid artery. (**B**) is a 48-year-old male. This is a DSA image of his left common carotid artery. The red arrows show the location of M1 stenosis.

The beginning segment of the middle cerebral artery (MCA) has localized stenosis with incomplete occlusion of the vessels distal to M1. Positive cases are assigned a value of 1, while negative cases are assigned a value of 0 (Figure 6).

#### 2.3.9. Factor 4 PCA Anomaly

**Figure 7:**
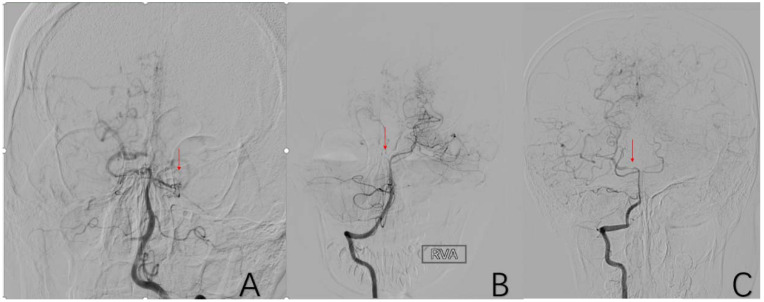
(**A**) is a 52-year-old female. Vascular occlusion of distal vessels from P1. (**B**) is a 6-year-old male with posterior cerebral artery initiation occlusion. (**C**) is an 8-year-old male who had localized stenosis in P1 with essentially normal distal vessel visualization. The red arrows in (**A**–**C**) are the tool of the PACS and show the location of PCA anomaly.

Abnormal pathology of the main trunk or branches of the posterior cerebral artery, such as stenosis, occlusion, and smoke-like angiogenesis. Positive cases are assigned a value of 1, while negative cases are assigned a value of 0 (Figure 7).

#### 2.3.10. Factor 5 Posterior Circulation Compensation

**Figure 8:**
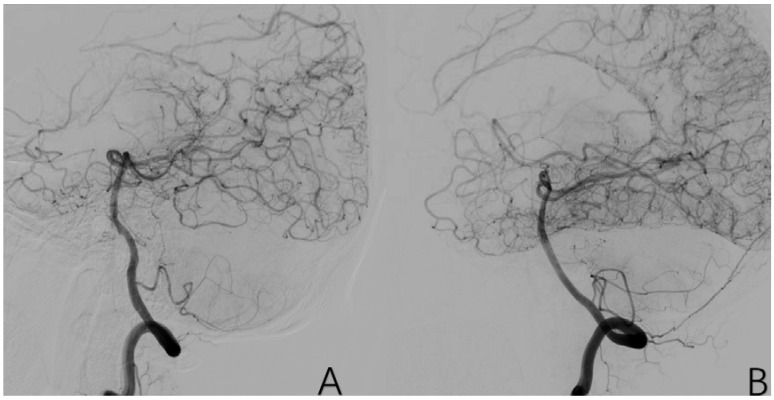
(**A**) is a 43-year-old male. (**B**) is a 32-year-old female. Both of them have typical posterior circulation compensatory imaging manifestations.

The PCA supplies blood to the original blood supply area of the MCA and the ACA through various traffic branches and anastomotic branches. Positive cases are assigned a value of 1, while negative cases are assigned a value of 0 (Figure 8).

#### 2.3.11. Factor 6 Unstable Compensation

**Figure 9:**
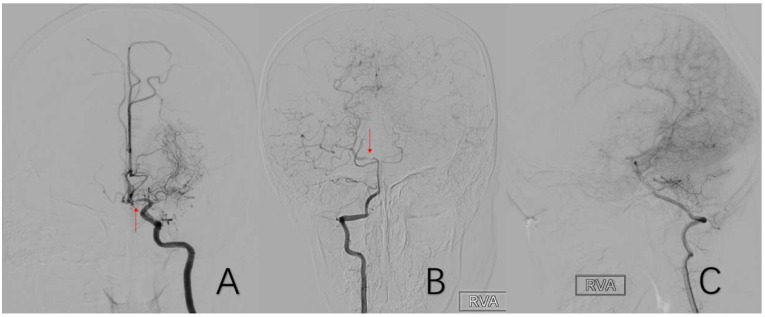
(**A**) is a 54-year-old male who had left A1 stenosis while supplying the right anterior cerebral artery A2 and distal branches vicariously through the anterior communicating artery. (**B**,**C**) are the same 8-year-old male. He had localized stenosis of the right P1 and a compensatory blood supply from the right posterior cerebral artery to the anterior cerebral artery and middle cerebral artery. The red arrow in (**A**) shows the location of A1 stenosis. The red arrow in (**B**) shows the location of P1 stenosis.

A vessel has an abnormal pathology, and at the same time, it must also compensatorily supply blood to other vascular supply areas. Positive cases are assigned a value of 1, while negative cases are assigned a value of 0 (Figure 9).

#### 2.3.12. Factor 7 Extracranial Arterial Compensation

**Figure 10:**
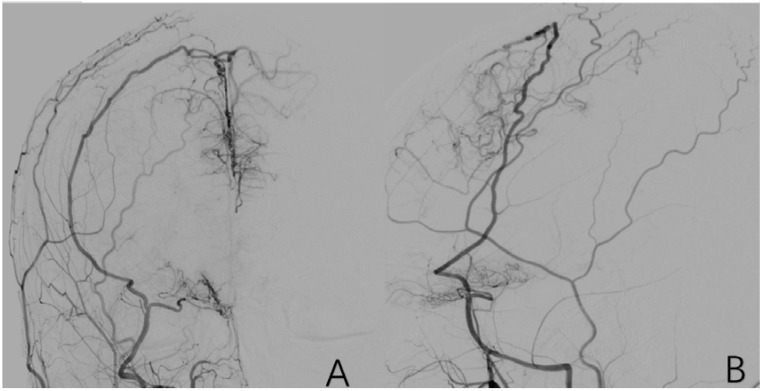
(**A**,**B**) is a 9-year-old female with a right-sided external carotid arteriogram showing compensatory blood supply from the right middle meningeal artery to the cranial area.

Various branches of the external carotid artery supply blood to the cranium in a compensatory manner by various means. The most common of these is the compensatory blood supply from the middle meningeal artery. Positive cases are assigned a value of 1, while negative cases are assigned a value of 0 (Figure 10).

### 2.4. Statistical Analyses

Postoperative cerebral infarction cases were defined as the experimental group, and cases without postoperative cerebral infarction were defined as the control group [12,25,26,27,28,29].

Statistical analysis was performed by the Pearson chi-square test, statistical analysis, analysis of variance (ANOVA), and multifactor logistic regression analysis [13,14,15,16,30,31].

For single-factor analysis, statistical analysis was performed using the Pearson chi-square statistic and Fisher’s exact test for binary variables. Analysis of variance (ANOVA) for continuous variables.

Multifactor logistic regression analysis was performed for predictors associated with postoperative cerebral infarction. In the multifactor logistic regression analysis, the method was as follows: forward: conditional, entry: *p* < 0.05, removal: *p* > 0.10. Twelve independent variables were analyzed. All statistical analyses were performed using SPSS software (Version 27.0). The cutoff point for statistically significant (*p* values < 0.05).

## 3. Results

A total of 326 CR cases of ischemic MMD were collected in this trial. There were 326 surgeries with 277 (85.0%) postoperative infarct-free (the control group) and 49 (15.0%) postoperative infarcts (the experimental group).

In the single-factor analysis, three general factors and five angioarchitectural factors were found to be related to postoperative cerebral infarction (*p* < 0.05), and they were age (*p* = 0.013), TIA (*p* < 0.001), old cerebral infarction (*p* = 0.002), ICA stenosis (*p* = 0.020), A1 stenosis (*p* < 0.001), M1 stenosis (*p* < 0.001), PCA anomaly (*p* < 0.001), and unstable compensation (*p* < 0.001).

Four variables were not significantly different between the two groups (*p* ≥ 0.05): gender (*p* = 0.360), side (*p* = 0.698), posterior compensation (*p* = 0.087), and extracranial compensation (*p* = 0.765) (Table 1).

In the multivariate analysis, three general factors and four angioarchitectural factors were associated with postoperative cerebral infarction. Age (OR, 0.969; 95%CI, 0.939–1.000; *p* = 0.049), A1stenosis (OR, 5.843; 95%CI, 1.730–19.732; *p* = 0.004), M1stenosis (OR, 6.206; 95%CI, 2.079–18.526; *p* = 0.001), PCA anomalies (OR, 4.367; 95%CI, 1.452–13.129; *p* = 0.049), Unstable compensation (OR, 5.335; 95%CI, 1.427–19.948; *p* = 0.013), TIA (OR, 4.264; 95%CI, 1.844–9.863; *p* = 0.001), and Old cerebral infarction (OR, 2.972; 95%CI, 1.194–7.397; *p* = 0.019). The above seven factors can be used in the regression equation to predict the probability of postoperative cerebral infarction (Table 2).

The result of the multifactor logistic regression analysis is Z = −4.392 to 0.032 (Age) + 1.765 (A1 stenosis) + 1.825 (M1 stenosis) + 1.474 (PCA anomalies) + 1.674 (Unstable compensation) + 1.450 (TIA) + 1.089 (old infarction), and goodness of fit is 90.2%, which means the accuracy in predicting probability is 90.2%. Probability of postoperative cerebral infarction *p* = e^z^/(1 + e^z^). The values in each factor were used in the result of multifactor logistic regression analysis. For example, the probability of postoperative cerebral infarction in a 30-year-old MMD patient with A1 stenosis and no other risk factors is calculated as follows: Z = −4.392 to 0.032(30) + 1.765(1) + 1.825(0) + 1.474(0) + 1.674(0) + 1.450(0) + 1.089(0). Z = −1.667. *p*= e^z^/(1 + e^z^) = 15.88%.

## 4. Discussion

Individualized treatment plans are increasingly being advocated for because of the perceived differences in patient preoperative presentation and imaging performance [17,18,20,32,33,34]. Therefore, determining the appropriate surgical approach based on preoperative conditions and reducing the chances of postoperative complications have been central issues in designing treatment plans. Similar to our daily work experience, here, we found that age, TIA, and old cerebral infarction were all associated with postoperative cerebral infarction. These variables suggest that the patient’s ability to tolerate cerebral ischemia is poorer, the cerebrovascular surrogate blood supply is poorer, and the chances of postoperative cerebral infarction are increased. TIA suggests that the intracerebral vessels are in a decompensated stage and that the intracerebral blood supply is unstable and more prone to cerebral infarction. The effect of age on postoperative cerebral infarction has been studied extensively. Adult patients are more likely to have postoperative cerebral infarction than pediatric patients [19,23,25,35,36,37,38]. The cause of ischemic stroke is partly attributed to progressive occlusion of the main cerebral arteries. Because the development of collateral vessels (moyamoya vessels) is generally not significant in adults, sudden occlusion may cause more severe consequences in adults than in pediatric patients. Therefore, the immediate increase in cerebral blood flow may have a more detrimental effect on the surgical hemisphere in adults than in pediatric patients. However, direct and combined procedures have been increasingly used because the use of indirect procedures for revascularization often results in inadequate revascularization in adults.

Posterior circulation compensation and extracranial vascular compensation play an important role in the treatment of patients with MMD, and the results of this study suggest that these two factors do not significantly improve the odds of postoperative cerebral infarction. A possible reason for this is that we overestimated the role of these two factors during preoperative evaluation, assessed a proportion of such patients as high risk, and therefore used a safer indirect revascularization, which disrupted the distribution of such patients and caused a bias in the data analysis [9,23,39,40,41]. Therefore, these two variables should not be ignored during preoperative evaluation.

Five angioarchitectural factors were central to this study. They are all closely related to postoperative cerebral infarction. They all share a common manifestation, namely, an unstable hemodynamic state. For many patients with MMD, the pathological changes are chronic and long term, and the brain tissue corresponding to the lesion will develop corresponding ischemic tolerance mechanisms or compensatory mechanisms, such that the brain tissue is better adapted to the environment of insufficient cerebral blood supply. In contrast, in the unstable hemodynamic state, there are abnormal changes in the proximal vessels of cerebral arteries, while the distal vessels or branch vessels are not completely occluded. As a consequence, the distal brain tissues do not have ischemic tolerance mechanisms formed by long-term ischemia and are more sensitive to changes in the cerebral blood supply and more prone to cerebral infarction [9,19,23,33,37,38,39,40,41,42].

Some studies have found that postoperative infarction may also be associated with acute occlusive changes in the ACA, MCA, and PCA. It is clear that in an unstable hemodynamic state, the probability of such acute occlusion will be greatly increased.

Therefore, it has been suggested that a detailed assessment of hemodynamics in the perioperative and general anesthesia states is essential. This includes the levels of PaCO_2_, hematocrit, circulating volume, blood pressure, and cerebral perfusion pressure, all of which may be altered, which in turn may cause changes in the cerebral blood supply and be the cause of postoperative cerebral infarction in patients. Angioarchitectural factors are closely related to hemodynamic blood flow and have a direct impact on hemodynamic alterations in patients [15,16,17,31,32,33].

In this study, we found that we could combine these seven variables to accurately predict the odds of cerebral infarction after a combined surgical approach. One study found that with a well-established preoperative evaluation, the probability of postoperative cerebral infarction was not significantly higher than that with the indirect surgical approach, despite the difficulty and time required to complete the anastomosis with either the direct or this combined surgical approach. We attribute this difference to the immediate increase in cerebral blood flow after surgery. The immediate increase in blood flow with direct or combined revascularization may compensate for the adverse effects often observed postoperatively, such as weeping-induced hypocapnia, hypotension, and loss of circulating volume. In contrast, indirect surgery alone may reduce cerebral blood flow in the acute postoperative period. Thus, patients with severely impaired hemodynamics are at risk for postoperative systemic ischemia [13,14,15,16].

This study shows that if MMD follows the seven factors of patient age ≥ 43, TIA ≥ 1 within 6 weeks prior to surgery, maximum diameter of infarct lesion ≥ 10 mm, ICA stenosis, A1 stenosis, M1 stenosis, PCA anomaly, and unstable compensation, postoperative cerebral infarction is likely. The morbidity can be predicted using the following equation: Z = −4.392 − 0.032 (age) + 1.765 (A1 stenosis) + 1.825 (M1 stenosis) + 1.474 (PCA anomalies) + 1.674 (unstable compensation) + 1.450 (TIA) + 1.089 (old infarction). Its goodness of fit is 90.2%, which means it has a very high accuracy in predicting probability. The probability of postoperative cerebral infarction is P = e^z^/(1 + e^z^). 

### Limitation

First, all patients were registered at a single center, and potential selection bias for region and ethnicity may be present.

Second, this study did not include intraoperative changes such as duration of anesthesia, PaCO2, hematocrit, circulating volume, blood pressure, and level of cerebral perfusion pressure.

Third, there is no directly quantifiable way to evaluate flow dynamics changes, which can only be indirectly inferred from DSA performance.

Fourth, our study was retrospective; therefore, further studies are needed to evaluate its value in a prospective manner.

## 5. Conclusions

Age, TIA, old infarction, and four angioarchitectural factors, including A1 stenosis, M1 stenosis, PCA anomaly, and Unstable compensation, can be taken to quantify the probability of postoperative cerebral infarction in ischemic MMD following combined revascularization. The point values for each factor can be seen in Section 2.4 Data Collection. 

## Figures and Tables

**Table 1:** Single-factor analysis results.

Total (N = 326)		No Infarction (N = 277)	Infarction (N = 49)	Value	*p*
Gender	Male	133(83.1%)	27(16.9%)	1	0.360
	Female	144(86.7%)	22(13.3%)	2	
Side	Left	133(84.2%)	25(15.8%)	1	0.698
	Right	144(85.7%)	24(14.3%)	2	
Age	Mean age	37.6 ± 13.9	42.8 ± 11.3	integer of age	0.013
TIA	Positive	33(61.1%)	21(38.9%)	1	<0.001
	Negative	243(89.7%)	28(10.3%)	0	
Old infarction	Negative	127(92.0%)	11(8.0%)	0	0.002
	Positive	150(22.4%)	38(77.6%)	1	
ICA stenosis	Negative	273(85.8%)	45(14.2%)	0	0.020
	Positive	4(50%)	4(50%)	1	
A1 stenosis	Negative	266(87.8%)	37(12.2%)	0	<0.001
	Positive	11(47.8%)	12(52.2%)	1	
M1 stenosis	Negative	265(88.0%)	36(12.0%)	0	<0.001
	Positive	12(48.0%)	13(52.0%)	1	
PCA anomaly	Negative	264(88.3%)	35(11.7%)	0	<0.001
	Positive	13(48.1%)	14(51.9%)	1	
Posterior compensation	Negative	50(78.1%)	14(21.9%)	0	0.087
	Positive	227(86.6%)	35(13.4%)	1	
Unstable compensation	Negative	271(88.0%)	37(12.0%)	0	<0.001
	Positive	6(33.3%)	12(66.7%)	1	
Extracranial compensation	Negative	221(84.7%)	40(15.3%)	0	0.765
	Positive	56(86.2%)	9(13.8%)	1	

**Table 2:** The results of multifactor factor analysis.

	B	*p*	OR	95% CI for OR	
				Lower	Upper
Age	−0.032	0.049	0.969	0.939	1.000
A1 stenosis	1.765	0.004	5.843	1.730	19.732
M1 stenosis	1.825	0.001	6.206	2.079	18.526
PCA anomaly	1.474	0.009	4.367	1.452	13.129
Unstable compensation	1.674	0.013	5.335	1.427	19.948
TIA	1.450	0.001	4.264	1.844	9.863
Obsolete infarction	1.089	0.019	2.972	1.194	7.397
Constant	−4.392				

B: coefficient of the variable. *p*: significant result. OR: odd ratio. CI: confidence interval. PCA: posterior cerebral artery. TIA: transient ischemic attack.

## Data Availability

All data generated or analyzed during this study are included in this published article.
